# Natural Products to Fight Cancer: A Focus on *Juglans regia*

**DOI:** 10.3390/toxins10110469

**Published:** 2018-11-14

**Authors:** Elena Catanzaro, Giulia Greco, Lucia Potenza, Cinzia Calcabrini, Carmela Fimognari

**Affiliations:** 1 Department for Life Quality Studies, Alma Mater Studiorum-University of Bologna, corso d’Augusto 237, Rimini 47921, Italy; elena.catanzaro2@unibo.it (E.C.); giulia.greco9@unibo.it (G.G.); cinzia.calcabrini@unibo.it (C.C.); 2 Department of Biomolecular Sciences, University of Urbino Carlo Bo, via Saffi 2, Urbino 61029, Italy; lucia.potenza@uniurb.it

**Keywords:** *Juglans regia*, walnut, cancer therapy, in vitro studies, in vivo studies, natural products

## Abstract

Even if cancer represents a burden for human society, an exhaustive cure has not been discovered yet. Low therapeutic index and resistance to pharmacotherapy are two of the major limits of antitumour treatments. Natural products represent an excellent library of bioactive molecules. Thus, tapping into the natural world may prove useful in identifying new therapeutic options with favourable pharmaco-toxicological profiles. *Juglans regia*, or common walnut, is a very resilient tree that has inhabited our planet for thousands of years. Many studies correlate walnut consumption to beneficial effects towards several chronic diseases, such as cancer, mainly due to the bioactive molecules stored in different parts of the plant. Among others, polyphenols, quinones, proteins, and essential fatty acids contribute to its pharmacologic activity. The present review aims to offer a comprehensive perspective about the antitumour potential of the most promising compounds stored in this plant, such as juglanin, juglone, and the ellagitannin-metabolites urolithins or deriving from walnut dietary intake. All molecules and a chronic intake of the fruit provide tangible anticancer effects. However, the scarcity of studies on humans does not allow results to be conclusive.

## 1. Introduction

The Italian musician Niccolò Paganini narrated the story of some Italian witches who used to practice the Sabbath around a mystic, majestic, deciduous tree in Benevento. Not without reason, that tree was a walnut tree. Since ancient times, the beneficial but also threatening properties of the walnut tree were celebrated. The fact that no other plant was able to grow close to that tree fostered an aura of intrigue and enigma around it. Later on, it was discovered that juglone, a molecule stored in kernels [1], leaves [2], and green husks [3] of *Juglans regia*, can be toxic for many plant species, impeding their growth in its vicinity [4]. Pliny the Elder attributed to the walnut tree magical and supernatural properties, and in his *Naturalis Historia*, renewing the tree as a deadly threat, he warned not to lie in its shade. In Greece, the tree was protected by Artemis, goddess of nature and the moon, who, according to the myth, built a temple from walnut wood in honour of Caria, who turned into a walnut tree from Dionysus. And so on, throughout history there are uncountable traces of walnut tree use, between myth and reality. From 4000 B.C., the walnut tree has been cultivated, mostly for its therapeutic and nutritional properties, and nowadays, it is exploited for its wood and fruit all-over the world [5].

The common walnut (*Juglans regia* L.), known also as English or Persian walnut, is a member of the *Juglandaceae* family. Due to their chemical compositions, many parts of the plant are used for their demonstrated biological activity; the nut (dry seeds or fruit), epicarp, shell, root, bark, leaves, and also green walnuts are full of bioactive compounds [3]. From ancient times, walnuts were not merely consumed as food, but different parts were used in folkloristic medicine, and at a later time, in the pharmaceutical and cosmetic industries [3]. Experimental and epidemiological studies clearly show that regular consumption of walnut seeds (from now on referred to as “walnut”) is beneficial for many chronic diseases, such as coronary heart disease [6], rheumatisms [7], diabetes [8,9,10], obesity [8], and cancer [10]. Furthermore, many studies show a correlation between regular walnut intake and reduced incidence of cancer [11]. Indeed, walnuts are an admirable source of nutrients and phytochemicals which are of great benefit for human health, such as polyphenols, proteins, fibers, sterols, and essential fatty acids [2]. Considering only the levels of phenolic antioxidants, walnut is the most enriched among nut species [12].

Natural products have a diversified composition comprising active molecules that will translate into a multi-target way of action. Indeed, phytochemicals stored in the plant can act in a synergistic way to concur a final biological effect. For this very reason, the American Food and Drug Administration (FDA) acknowledges natural products, the so-called botanical drugs, as therapeutic agents composed by “vegetable materials, which may include plant materials, algae, macroscopic fungi, or combinations thereof”. They are presented as “products that are marketed as diagnosing, mitigating, treating, or curing a disease”, and are thus deemed to be on an equal footing with traditional drugs in terms of activity and prescription regulations [13]. To date, two botanical drugs have been approved for marketing as prescription drugs, and several others as over-the-counter drugs, underlining the efficacy of this new category of therapeutic agents [13].

Among many diseases, the intrinsic nature of natural products and botanical drugs perfectly fits the complexity of cancer pathology. Cancer is still a major burden all over the world. Although many anticancer agents have been identified, toxic effects and resistance impose as huge limits on their use [14]. Indeed, in the majority of cases, the side effects of traditional drugs hamper clinical outcomes. Thus, the need for new agents characterised by better pharmaco-toxicological profiles is compelling. For a long time, reductionist approaches using isolated molecules dominated in cancer drug discovery [15]. However, an understanding of the therapeutic potential of botanical-drugs opened the way to explore the synergistic or additive effects that arise from the mixtures of compounds occurring in plants [15]. Furthermore, natural products are usually characterized by a better toxicological profile compared to traditional drugs. Thus, their use is conceived to increase the efficacy of traditional anticancer agents, and at the same time, to decrease their toxicity. Indeed, the combination of botanical and traditional agents would potentially decrease the dose of a given traditional drug that is necessary to reach the therapeutic effect, hence limiting its harmfulness [16].

The purpose of the present review is to give an update on the role of *Juglans regia* in cancer therapy. In vitro, in vivo, and clinical studies will be discussed, taking into account scientific works involving the single molecules found in the walnut and walnut extracts from different parts of the plant. The ability of walnuts to prevent some types of cancer will be discussed, but its antioxidant and anti-inflammatory property will not be explored in depth, since these properties are present in most, if not all plants. Moreover, oxidative stress and inflammation play a role in the pathogenesis of many diseases, and not only of cancer, and any discussion thereof would go beyond the aims of this review.

## 2. Phytochemical Profile of *Juglans regia*

*Juglans regia* L. is a deciduous tree belonging to the *Juglans* genus (family *Juglandaceae*) [17]. It has short trunk and a wide crown, deep roots, with a substantial tap root which develops during the juvenile stage [18,19]. The bark is dark grey with wide fissures. Compound alternate leaves consist of 3–5 pairs of elongate-ovate, slightly crenate, apical acuminate leaflets [20]. Male flowers are 6-lobed, show 12–18 stamens and drooping catkins with lanceolate bracts. Female flowers are formed by a sessile bilobed stigma and by the ovary; they are single or grouped into 2 or 5 terminal clusters [21]. The fruit ripens during the hottest summer period. It is a rounded nut of 4–5 cm and weighing up to 18 g; it has a greenish husk (mesocarp), a shell (endocarp), and a large kernel [22]. There is high variability between shape, size, weight of fruits, colour, and thickness of shell and size, weight and external colour of kernel, which is ascribable to genetic variability of walnuts from different regions of the world [23]. The edible part of the plant is the seed or kernel, and is consumed fresh, toasted, or used as a part of other edible products. *Juglans regia* may be propagated both by seeds and vegetatively.

This species survived the last glaciation and the large number of *Juglans regia* with phenotypic diversity that are found in Asian countries such as Kyrgyztan, Uzbekistan, and Tajikistan has led prevalent opinion to consider central Asia as the primarily place of the genus’ origin and diversity [24]. Trade and cultural expansion allowed walnut dispersion into new habitats, including Europe, north Africa, east Asia, the USA, and South America. Earliest cultivation has been documented by pollen records in the Mediterranean area, in particular in Italy ca. 6000 years ago, in north-eastern Greece, Croatia, and Anatolia ca. 4000 years ago [25]. Nowadays, *Juglans regia* distribution ranges between 10° and 50° northern latitude and requires specific climatic conditions, such as hot and sheltered regions and a long season of growth. Germination is improved in mild winters [19,26].

The kernels contain unsaturated fatty acids, polyphenols, available carbohydrate, proteins, fiber, vitamins (vitamins E, B6, niacin, folic acid), phytosterols (stigmasterol, campesterol, sitosterol), and minerals (copper, magnesium, potassium). They are a nutrient-dense food, largely due to their fat (about 60% in freshly weight) and protein content [27,28]. Neutral lipids are the main oil components (96.9% of total lipids), especially triacylglicerides. Low quantities of polar lipids mainly consist of sphingolipides. Triacylglycerides are rich in oleic (18:1 n-9), linoleic (18:2 n-6) (LA) and linolenic (18:3 n-3) (LNA) acids. Thus, this fruit has a high concentration of ω-6 and ω-3 polyunsaturated fatty acids (PUFAs) [29]. The percentage of fatty acids in *Juglans regia* walnut oil varies widely. The oleic acid content of the oils ranges from 12.7% to 34%, LA ranges from 49.7% to 72%, LNA from 9% to 25%, saturated palmitic acid is between 5.24% to 8.2%, and stearic acid ranges from 1.4–2% to 5% [30,31,32]. Phenolic compounds found in the kernel are gallic acid, catechin, vanillic acid, cinnamic acid, pyrocatechin, epicatechin, rutin, syringic acid, and juglone [33]. Gallic acid and catechin are the most readily-bioavailable compounds. Juglone (5-hydroxy-1,4-naphtoquinone) is an oxygen derivative of naphthalene. In living plants, juglone is in a non-toxic glycosylated form, but when exposed to soil or air, this allelochempound is immediately transformed into an oxidized, highly toxic form. Colaric et al. found that the concentration of juglone in the fruit was significantly higher compared to other phenols [1]. The kernel pellicle of walnut, which represents only 5% of the kernel weight, is rich in ellagitannins (ETs) [34], and is a much better source of phenolic compounds than the kernel. Tellimagrandin I and tellimagrandin II are among the identified ETs [35]. After intake, ETs are hydrolyzed, producing ellagic acid (EA), which is metabolized by microbiota in urolithins-A (Uro-A) and -B (Uro-B). These are absorbed and may have different biological activities [36,37]. Walnut has been placed on the FAO list of crucial plants for its nutritive value [38].

The walnut green husk is a by-product of walnuts crops. The green husk from mature fruit is rich in polyphenolic compounds: chlorogenic acid, caffeic acid, ferulic acid, sinapic acid, gallic acid, EA, protocatechuic acid, syringic acid, vanillic acid, catechin, epicatechin, myricetin, juglone [39]. Cytotoxic diarylheptanoids juglanin A and juglanin B, rhoiptelol, and an alpha-tetralone derivative (regiolone) have been also found [40].

Leaves contain tannins, naphtalene derivatives, flavonoids, phenolic acids, volatile oils, and other substances, including acid ascorbic, cyclitols, mucilage, calcium, potassium, and ash. Among tannins, approximately 10% is EA. Juglone is the most known naphthoquinone, which occurs in fresh leaf [41,42]. Significant amounts of juglone were found, i.e., in the range of 13.1–1556.0 mg/100 g dry weight, in 1121 samples of leaf [43]. The highest content of phenolics in leaves was found in May and July.

The content of phenolics in different parts of walnut depends on many environmental conditions, genotype of different cultivars [1,44] and sampling date [44,45]. For instance Cosmulescu et al. reported that walnut leaves have higher phenolic content in July and early September, with a decrease in August [45].

## 3. Anticancer Activity

### 3.1. In Vitro Studies

**Figure 1:**
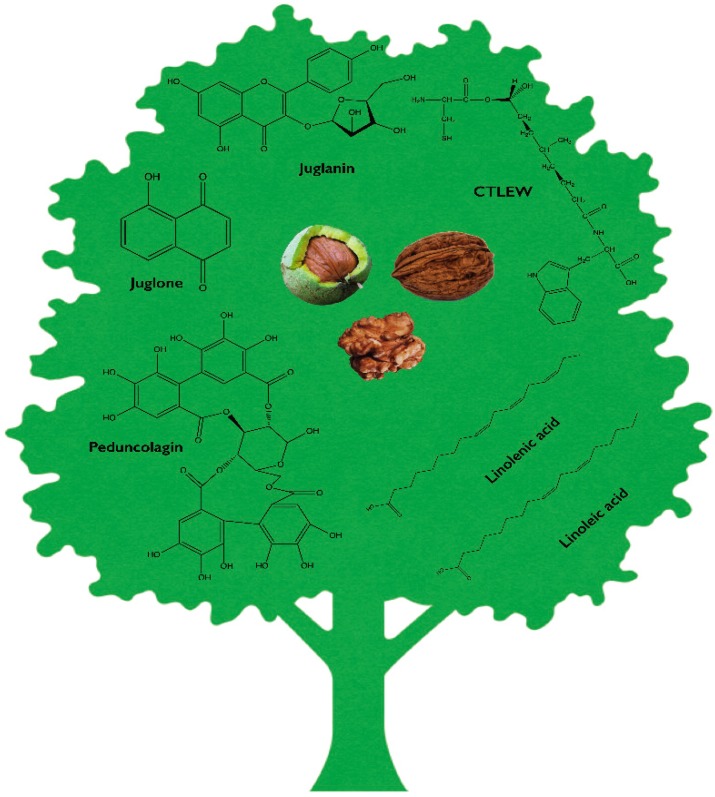
The most characterised bioactive compounds of *Juglans regia*.

As above illustrated, walnut trees store numerous and very diversified nutrients and phytochemicals (Figure 1). Many constituents have been successfully isolated and their antitumour potential investigated. Indoles, polyphenols such as tannins and flavonoids, proteins, and fatty acids, alone or in combination, have been identified as promoters of the health benefits ascribed to walnut consumption [46]. The following paragraphs will present in vitro evidence for the most characterised compounds and extracts from different parts of the plant.

#### 3.1.1. Uro-A

Walnuts represent the seventh main source of polyphenols among the studied food and beverages [12]. ETs are the most studied representatives of this class of compounds, and among them, pedunculagin (PDN) (Figure 1), found in *Juglans regia*, shows the greatest abundance [47]. As all ETs, PDN is subject to metabolic reactions in mammals. The first hydrolytic step foresees the release of EA, which moves to the gut flora where, through a second metabolic transformation, generates biologically-active molecules known as Uros. After ETs consumption, the resulting Uros are free to circulate in the blood flow where they reach micromolar levels (about 100 μM after 24–48 h), and place themselves into organs in both free or conjugated form [48]. Uros distribute in plasma, urine, and faeces, and in humans, they can be found in some organ tissues, such as the prostate gland [49], or colon [50].

As a consequence of different gut microbiota compositions, the quality and quantity of Uros produced varies considerably among individuals. However, many studies demonstrated that the anticancer activity of walnut lies on two isoforms of Uros (Uro-A and IsoUro-A) [51,52] that, coupled with Uro-B, are the most abundant ET-derived metabolites in the gut of mammals and humans after walnut intake [53,54]. For this reason, only the anticancer potential of Uro-A and Uros mixtures will be discussed in this review.

The anticancer potential of Uros relies on proliferation inhibition, apoptosis induction, and other tumour-specific mechanisms.

Taking into account the fact that Uros are produced in the gastrointestinal tract, and that thanks to the enterohepatic recirculation, they persist in the colon for long time [53], many studies were conducted on in vitro models of colon carcinomas. Many works reported the ability of Uros and a mixture of Uros and ETs to inhibit cell proliferation in a cell line-dependent manner. It has been demonstrated that EA and many Uros tested alone (Uro-A, -B, -C, IsoUro-A) at an arbitrary concentration of 100 μM were able to inhibit cell proliferation. Uro-A, Uro-C and IsoUro-A showed the greatest effect. In fact, only these three compounds were able to induce the double G2/M and S proliferation block, while the others caused only a slight S phase arrest [52]. In 2009, Gonzàlez-Sarrías et al. [55] reported that Uro-A, and, to a higher extent, a mixture of Uros and ETs, inhibited Caco-2 cell proliferation via G2/M arrest, with the involvement of cyclin B1, while Kasimsetty et al. [56] reported that Uro-A inhibited HT-29 cell proliferation via G0/G1 and G2/M arrest, tailed by apoptosis induction. A recent study on Caco-2, HT-29, and SW480 cells confirmed the ability of Uros to inhibit cell proliferation. In this case, the study analyzed the effects of the actual average content in Uros and EA found in faeces and colon after walnut consumption in humans according to the main macro metabotypes identified (Table 1). Depending on how individuals metabolise ETs, they qualify as Uro-A producers (Uro-A is the main metabolite produced), Uro mixture producers (Uro-A is quantitative similar to Iso-Uro-A and Uro-B), or Uro null that includes all the rest of the population [57]. The first tested-mixture (mA) was enriched in Uro-A (85 μM Uro-A, 10 μM Uro-C, and 5 μM EA), while the second (mB) was enriched in IsoUro-A and Uro-A (50 μM IsoUro-A, 30 μM Uro-A, 10 μM Uro-B, 5 μM Uro-C, and 5 μM EA). In SW480 and Caco-2, the mixtures had comparable behaviour, while with HT-29, the effect was lower [52]. The high glucuronidation rate of Uros in HT-29 cells (much higher than in Caco-2 and SW480) could explain this behaviour [58]. Both mixtures, mA and mB, equally blocked the cell cycle in G2/M and S phase, and induced apoptosis immediately afterwards. At the molecular level, Uros (tested alone and together) modulated different microRNAs (miRNAs), but how this effect was linked to their antitumour potential is not completely understood [52]. MiRNAs are short sequences of nucleotides (21–25) that are not able to code for RNA, but are involved in the post-transcritptional regulation of gene expression. For example, they are able to repress gene post-transcription or degrade the mRNA to which they are bonded [59]. In the three cell lines, miRNAs and gene expression were differently modulated, suggesting that the molecular cascade driving the antiproliferative effects of Uros is cell-type specific. In Caco-2 cells, single Uros and to a greater extent mixtures downregulated miR-224, that in turn provoked an increased gene expression of p53 and the cyclin-dependent kinase inhibitor 1A (CDKN1A). CDKN1A is responsible for the transcription of p21 [52], a crucial cyclin-dependent kinase inhibitor that blocks the cell cycle [60]. In HT-29, a downregulation of miR-215 was observed, while MiR-224 was not modulated. No significant change for any of the two miRNAs was recorded in SW480 cells [52].

The role of autophagy in the maintenance of cellular homeostasis and protection for stressed cells is well understood, while its potential as an anticancer mechanism still needs to be studied. Autophagy can elicit a non-apoptotic form of programmed cell death that is potentially useful for tumours that are resistant to apoptosis [61]. Apoptosis is, by definition, a form of regulated cell death, while until few years ago autophagy was considered a cellular defense mechanism against cell death. Now, it is clear that autophagy can exert either pro-survival or pro-death stimuli. Furthermore, it can synergise apoptosis or replace it when apoptosis is suppressed [62,63]. Interestingly, the anticancer potential of Uro-A lies also in autophagy promotion. At sub-micromolar levels (starting at 1.5 μM), it promoted autophagy in a human SW620 cell line that persisted until 30 μM, when apoptosis and cell-cycle inhibition occurred [64]. Like many polyphenols, the ability of Uro-A to provoke autophagy, apoptosis [61], and cell-cycle inhibition [65] maximises the possibility to completely eradicate tumour cells via multiple pathway activations. In the same cell line, Uro-A downregulated the expression of matrix metallopeptidase 9 (MMP-9), a protein directly linked to tumour invasion and metastasis [64].

To comprehensively examine the interesting pharmacological profile of Uros on colon cancer cells, we include some studies reporting its ability to sensitise cancer cells to traditional anticancer drugs, and to selectively target cancer cells. At 20 and 40 μM, Uro-A increased sensitivity of Caco-2, SW480, and, to a lesser extent, HT-29 cells to 5-fluorouracile (5FU) and its metabolite 5-deoxy-5-fluorouridine, which translated in a decreased 50% inhibitory concentration (IC_50_) of those drugs, in a significant accumulation of cells in S and G2/M phases caused by the accumulation of cyclin B1 and A, and in a slight activation of the apoptotic machinery [66]. Uro-A exhibited a remarkable safety profile against normal cells. Mixtures and single Uros showed a less marked effect on the non-transformed cell line CCD18-Co than that recorded on tumour cells, suggesting a selectivity of action towards cancer cells [52].

On two different models of hepatocellular carcinoma, HepG2, and epatitis B virus-transfected HepG2 (HepG2.2.15), Uro-A blocked cell proliferation and invasion through different mechanisms [51,67]. Reduced mitogen-activated protein kinase p38 (p38-MAPK) levels and overexpression of mitogen-activated protein kinase 1 (MEKK1) and c-Jun are usually found in growing malignant tumours. The P38-MAPK signaling cascade is involved in apoptosis elicitation in different ways. C-Jun regulates the transcriptional levels of cyclin D1 and, alone or in combination with p38-MAPK-altered signal, represses p53 transcription [68,69], thereby allowing uncontrolled cell-cycle progression. Beside the p38-MAPK signaling cascade, Wingless (Wnt) proteins control cell proliferation and cell self-renewal, behaving as inter-cellular signal transmitters. Extracellular stimuli transfer signals into the cell through cell surface receptors, and activate one of the several intracellular Wnt signal transduction cascades, such as the Wnt/β-catenin-dependent pathway or β-catenin-independent pathway [70]. The improper activation of the downstream cascade translates in the seamless promotion of cell proliferation, tumour growth, malignant invasion, and metastasis [51]. For instance, accumulation or mutation of β-catenin promotes the nuclear activation of T-cell factor/lymphoid enhancer-binding factor (Tcf/Lef) and the ensuing transcription of Wnt signalling target genes, such as cyclin D1 or c-Myc [51]. On one hand, in HepG2 cells, Uro-A promoted p38-MAPK activation, significantly suppressed c-Jun phosphorylation (i.e., activation), and decreased β-catenin expression. As a consequence, it enhanced cyclin D1 and c-Myc levels and p53 phosphorylation, contributing to prompt apoptosis [51]. On the other hand, in HepG2.2.15 cells, Uro-A targeted the Lin28a/let-7a axis. Let-7 belongs to a miRNA family involved in tumour suppression. Thus, its loss is associated with many types of tumours [71]. Let-7 can be repressed by Lin28, a primary transcript inhibitor of let-7 [72,73]. One function of let-7 is inhibiting high mobility group A (HMGA) and K-ras [74,75] proteins, involved in cellular epithelial-mesenchymal transition (EMT), oncogenic transformation, and metastatisation [76,77]. Uro-A elevated let-7 levels, while suppressing Lin28a, HMGA, and K-ras expression [67].

The activation of phosphatidylinositol-4,5-bisphosphate 3-kinase (PI3K)/protein kinase B (Akt) allows defective cells to escape cell-cycle checkpoint surveillance, avoiding apoptosis. In two tested bladder cancer cell lines, Uro-A elicited cell-cycle inhibition and apoptosis. In UMUC3 cells, apoptosis was attributed to the blockage of PI3K/Akt pathway [78], while on T24 cells, the activation of caspase 3 was mediated by p38/MAPK and c-Jun cascades [79]. In normal BJ cells, the IC_50_ was more than 5 times higher than that observed in cancer cells [78], confirming the selectivity of Uros towards cancer cells.

Prostate, breast, and endometrial cancer have one thing in common: they can exhibit hormones receptors. Accordingly, androgens in prostate tumours and both estrogens and progesterone in breast and endometrial tumours are able to modulate the etiology and the progression of these diseases [80]. In particular, the activation of hormone receptors fuels cell growth and proliferation, as well as tumour initiation and progression. Thus, in the case of hormone receptor positive prostate, breast, and endometrial cancer, hormone ablation therapy or hormone receptor modulations are very efficient therapies [81]. Uro-A exerted cytotoxic effects against androgen receptor positive (AR+) LNCaP, C4-2B and enzalutamide-resistant C4-2B prostate cancer cells [37,82,83]. These effects included cell-cycle inhibition in a p21-mediated way, and a consequent Bcl-2-induced apoptosis. In contrast, the effects on the AR negative (AR-) PC-3 cell line were limited, and the full cytotoxic effect occurred only if PC-3 cells were transfected to expose ARs [83]. Uro-A was able to downregulate prostate specific antigen (PSA) expression [37], a biomarker of prostate tumours that is also involved in tumour growth, invasion, and metastasis [84], and the expression and nuclear localisation of ARs. This evidence, taken together, suggests that Uro-A is an AR antagonist [82,83].

Uro-A has an affinity for estrogen receptors (ERs), but with a twofold effect. In the presence of estradiol, it showed antiestrogenic activity, while if estradiol was absent, it mimicked estrogenic activity [85]. Accordingly, Uro-A blocked ER+ MCF-7 cell proliferation only in an estradiol-enriched environment. In contrast, without any supplements, Uro-A promoted proliferation [86]. Uro-A was cytotoxic on ER- cell line (MDA-MB231), which suggests an estrogen-independent mechanism of action [87].

Finally, Uro-A suppressed the proliferation of HEC1A and Ishikawa endometrial cancer cells [88]. It stopped cell cycle at G2/M phase thanks to the modulation of specific cell-cycle proteins: increased expression of p21, cyclin B1 and E2, cell division cycle 25B (cdc25B), phosphorylated cyclin-dependent kinase 1 (p-cdc2), and myelin transcription factor 1 (Myt1). In parallel, Uro-A modulated the expression of estrogen-regulated genes via an ERα-dependent mechanism, thus acting as an estrogen agonist [88]. However, further studies are needed to determine how and if these effects are linked.

#### 3.1.2. Juglanin

An interesting polyphenol found in the green outer pericarp of walnut (green husk) is juglanin [40,126,127]. Juglanin (Figure 1) is a flavonoid and, as a member of this family, exhibits inhibitory activity against cancer growth, and promotion of inflammation response [128,129].

Juglanin demonstrated cytotoxicity on different models of breast (MCF-7, SKBR3, MDA-MB231 and BT474) [89], lung (A549, HCC827 and H1975) [90], and skin (UVB-stimulated B16F10) cancers. The *fil rouge* tracking juglanin’s antitumour potential is oxidative stress induction. Indeed, the juglanin-mediated ROS (reactive oxygen species) accumulation drove apoptosis and autophagy in lung and breast cancer cells [89,90]. In both cancer types, juglanin triggered both the extrinsic and intrinsic pathway of apoptosis. The extrinsic pathway involves the activation of death receptors at the cell surface, while different intracellular proapoptotic stimuli such as endoplasmic reticulum or oxidative stress are necessary to activate the intrinsic pathway. The latter pathway involves mitochondrial outer membrane permeabilisation (MOMP), and is controlled by the Bcl-2 protein family. Bcl-2 counts pro- and anti-apoptotic proteins, such as Bax and Bcl-2, respectively. Their ratio tips the balance towards cell survival or death. The characteristic mediators of both apoptosis pathways are caspases. The two cascades use different initiator caspases, such as caspase 8 for extrinsic or caspase 9 for intrinsic pathway, but they share common effector caspases (such as 3 and 7). Juglanin reduced the Bcl2/Bax ratio [89,90], activated caspases 3, 8 and 9 [89], and activated the tumour necrosis factor-related apoptosis-inducing ligand (TRAIL)/death receptors (DRs) relying on p53 activation [90]. The importance of triggering both pathways lies on the ability to promote apoptosis, even in the presence of defects in one of the two apoptotic machineries. This is a very common characteristic in tumour cells, and a major cause of drug resistance [130].

In juglanin-mediated antitumour activity, apoptosis and autophagy were triggered simultaneously, and exerted a synergistic activity [89,90]. Autophagy was probably mediated through the action of the autophagosome marker microtubule-associated protein light chain 3 (LC3), and the autophagy-regulating class III PI3-kinase (PI3K-III) and its Beclin 1-containing complex. Beclin-1/PI3K-III supports the enrollment of, and offers a platform for, crucial autophagy proteins engaged in the biogenesis of autophagosome [90].

Beside ROS, MAPKs are other targets of juglanin. C-Jun N-terminal kinase (JNK) is a member of MAPKs, whose main function is to regulate cellular proliferation, differentiation, and apoptosis. Whether JNK plays a pro-survivor or pro-death role relies on the inferred-stimuli and cell type involved in such activations [131,132]. In UVB-stimulated melanoma cells, the p38/JNK pathway is markedly attenuated. Juglanin exhibited antitumour activity triggering an anti-inflammatory cascade. Indeed, it was able to inactivate the PIK3/Akt pathway and suppress UVB-induced nuclear factor kappa-light-chain-enhancer of activated B cells (NF-κB) activation [91].

Juglanin synergised the effect of one of the most commonly-used antitumour drugs, doxorubicin (doxo). In particular, in both normal and doxo-resistant A549 cells, and normal and cisplatin-resistant H69 cells, it significantly increased the cytotoxic effect of doxo [133].

#### 3.1.3. Juglone

1,4-Naphtoquinones represent a class of molecules found in many plants, fungi, and bacteria which is characterised by an interesting anticancer profile [134]. The main mechanism underpinning their antitumour potential is the generation of semiquinone radicals and other ROSs in the endocellular environment [135]. Juglone (Figure 1) is a member of this family, acting as a growth-stunting agent [136] and apoptosis inducer. It exhibits antitumour activity on many types of tumours, such as breast [92,93,94,95,137], skin [99,100,137], glial cells [109,110,111,135,138], lung [137,138], prostate [105,106,107,137], pancreas [97], bladder [108], stomach [98], cervix [102,103,139,140], ovary [104], and blood [113,114]. For some of them, the mechanism of action has been investigated.

Juglone showed interesting antitumour potential in in vitro models of both androgen-dependent (LNCaP) and -independent (DU145) prostate cancers [106]. In LNCaP cells, it triggered apoptosis through the intrinsic pathway, promoting the activation of caspases 3 and 9, and decreasing mitochondrial potential (ΔΨ). At sub-toxic concentrations, it downregulated ARs and PSA expression [105], suggesting chemopreventive activity. Of note, different studies were conducted on the same cell line (LNCaP), but they did not agree about juglone’s potency. In one study, after 24 h, juglone showed an IC_50_ of 32.2 μM [107], while another at the same time obtained a double potency, evincing an IC_50_ of 13.8 μM [105]. An IC_50_ of about 15 μM was found from a third study on the same cell line, but after 48 h treatment [106], leading us to assume, for the sake of consistency, that the first and the third values are more reliable.

Cellular detachment from the tumour microenvironment, erosion of the contiguous extracellular matrix, and repositioning to a distal site, are the processes involved in tumour metastatisation. EMT allows epithelial cells to differentiate into mesenchymal, thereby letting them increase migration and invasiveness abilities that are linked to metastasis [141]. Androgen deletion therapy in prostate cancer can be followed by EMT [142]. Juglone upregulated the expression of the epithelial marker E-cadherin while reducing the mesenchymal factors N-caderin and vimentin. Furthermore, it synergistically inhibited the Akt/glycogen synthase kinase-3β (GSK-3β)/Snail axis that would physiologically promote E-cadherin repression and EMT induction [107].

On BxPC-3 and PANC-1 pancreatic cancer cells, juglone showed an IC_50_ of about 21 μM. At the same concentration, it lessened the adhesion features of pancreatic cells and decreased cell invasions by 56% and 80%, respectively, on BxPC-3 and PANC-1 cell lines. Juglone significantly dropped the protein level of MMP-9 and the vascular endothelial growth factor (VEGF) reporter Phactr-1 in both cell lines, while a drop of MMP-2 was evident only on BxPC-3 [97]. All these proteins are involved in tumour cell invasion. As reported above, the detachment of cancer cells from the original tumour, stroma invasion, vessel intravasation or extravasation to the target organ, and angiogenesis induction are the essential steps in metastatisation. The MMP family is a renowned metastasis marker since it plays a crucial role in tumour cell invasion through the digestion of several kinds of fibrillar extracellular matrix (ECM) elements, such as type IV collagen, i.e., the main constituent of the basement membrane [143,144,145]. Thus, juglone clearly demonstrated its beneficial activity on tumour metastasis and invasion.

Juglone exhibited anticancer effects on different breast cancer models. On MCF-7, doxo-resistant MCF-7 (MCF-7Adr) and transtuzumab-resistant SKBR3, juglone promoted G1 cell-cycle arrest [94,95] and ROS-driven apoptosis [92,93,95]. An exhaustive study on MCF-7 proved that juglone increased Bax/Bcl2 *ratio*, intracellular calcium (Ca^2+^) levels, ΔΨ disruption, cytochrome c (Cyt-c) release and caspase 3 activation, demonstrating the activation of the intrinsic apoptotic pathway [92]. On MCF-7 and SKBR3, it inhibited cell proliferation, colony formation, and migration capability [93,94], while on MCF-7Adr, angiogenesis was inhibited by decreased levels of VEGF-A, -B and -C [94]. The prooxidant profile of juglone was investigated on MCF-7. Glutathione (GSH), catalase (CAT), superoxide dismutase (SOD) and glutathione peroxidase protein levels diminished, validating the hypothesis of their consumption by juglone-induced ROS formation [93]. Juglone-mediated oxidative stress triggered forkhead box O3 (FOXO3), that, in turn, modulated p53 and altered the cellular homeostatic balance, prompting apoptosis [93]. Another interesting study showed that juglone cytotoxicity is, at least partially, ascribed to DNA damage. Indeed, juglone intercalated DNA in MCF-7 cells and caused oxidative cleavage [115]. However, no double strand breaks, in the form of the biomarker phosphorylated-H2A histone family member X (γ-H2AX), were recorded. In contrast, high levels of γ-H2AX were registered when juglone was tested in combination with ascorbate. As a matter of fact, juglone’s anticancer profile (in terms of proliferation inhibition, cytotoxicity, and ROS induction) was highly improved by ascorbate [115], revealing an interesting synergistic activity between these two compounds [93].

Juglone exhibited interesting effects also on human cervical cancer [102,103,139,140]. Already after 6 h, it promoted apoptosis on HeLa cells, inducing an upregulation of many proteins involved in the intrinsic and extrinsic pathway, such as Bax, Cyt-c, Fas cell surface death receptor (Fas), Fas-ligand. Moreover, it was found that JNK played a crucial role on juglone-mediated apoptosis [102]. The intrinsic pathway is also involved in juglone-induced apoptosis in the Caski and Siha cervix tumour models and SKOV3 ovarian cancer cells [104,139,140]. In the latter cell line, juglone induced G0/G1 cell-cycle arrest and inhibited invasion decreasing MMP-2 protein expression [104].

Melanoma represents 1% of all skin cancers, but it is the first in terms of deaths caused by skin tumours [146]. Juglone was able to kill melanoma cells in both normal (B16F1) [99] and resistant phenotypes (A2058 and MEWO) [100]. In all tested cell lines, juglone triggered apoptosis and oxidative stress. In B16F1 cells, it also consumed GSH [99]. In A2058 and MWEO, p53 and phspho-p38 levels were increased, suggesting their involvement in juglone-driven apoptosis [100]. Furthermore, on the latter cell lines, juglone significantly sensitised cells to the antitumour potential of TRAIL, which, when used alone, had a negligible effect. Furthermore, juglone confirmed its ability to promote DNA damage, as revealed by the significant increase in micronuclei frequency on B16F1 cells [99].

Juglone is a lipid-soluble molecule that may easily get through the blood-brain barrier; thus, its antitumour activity on glioblastomas was investigated. The interesting anticancer activity of juglone is applicable to different glioblastoma models, such as rat F-98 [135], human C6 [109,110], U251 [111], and U373 [138]. Different studies have shown its ability to promote cancer cell death on C6 glioblastoma multiforme cells that represent the most common and lethal tumours of the central nervous system. Juglone blocked cell proliferation and induced G0/G1 cell-cycle arrest on those cells, but not on normal glial cells [109,110]. As mentioned above, ROS production represents the foundation of juglone activity, and a recent work demonstrated that juglone-promoted oxidative stress is, at least in part, the result of the mitochondrial respiratory chain (MRC) complex I impairment [110]. MRC produces ATP, taking advantage of redox gradients. In particular, it is composed of four membrane-bound protein complexes (I–IV). They point to the production of an electro-chemical proton gradient throughout the inner mitochondrial membrane, which prompts oxidative phosphorylation in a way that the electron transfer is linked to the final complex ATP synthase, which produces ATP [147]. Of note, electron outflow can occur all along the MRC, and lead to ROS release and cell death [148]. For instance, complexes I, III, and IV are proton pumps that generate the mitochondrial membrane potential. An enduring fall or rise of mitochondrial membrane potential levels may induce an unwanted loss of cell viability. Complexes I–III are the main source of ROS; thus, the inhibition or impairment of these complexes may lead to ROS leakage [149]. Through high-resolution respirometry experiments, Sidlasuskas et al. [110] demonstrated that juglone decreased oxygen consumption mostly by disturbing the mitochondrial respiration mediated by complex I substrates (pyruvate/malate and glutamate/malate). Furthermore, amytal, a complex I inhibitor, decreased juglone-induced ROS production and reduced its anticancer activity [110], confirming the proposed mechanism of action.

Moving forward, juglone reduced spheroid invasiveness and contrasted the formation of metastases in the same cell line (C6) [109]. On U251 glioblastoma cells, juglone arrested cell growth by promoting apoptosis with the involvement of peptidyl-prolyl *cis*/*trans* isomerase (Pin1) inhibition [111]. Pin1 is an enzyme that regulates many cellular events, such as proliferation [150], neurons survival [151], differentiation [152], and metabolism [153]. Pin1 triggers numerous oncogenes or growth activators and also hinders many tumour suppressors or growth inhibitors [154]. Thereby, Pin1 ablation prevents cell growth, or affects various events like drug sensitivity, cellular motility, and metastasis formation [155]. Juglone is a well-known Pin1 inhibitor, and the study reported above directly linked Pin1 inhibition activity to juglone-mediated cytotoxicity. In the same cell line, juglone also impeded both cell migration and angiogenesis [111].

Cancer stem cells (CSCs) are a peculiar, small population of cancer cells. Like normal stem cells, CSCs are involved in tissue growth and repair; thus, their activity supports the development and progressive expansion of tumours. CSCs are able to initiate and propagate full-blown malignancy, contributing to therapeutic resistance and relapses [156]. Glioma stem-like cells (GSCs) can be targeted to favour better therapeutic outcomes. GSCs were obtained from U87 and two primary cell cultures (SHG62 and SHG66) using a serum-free medium supplemented with growth factors. Juglone-treated GSC experienced ROS-p38-driven apoptosis [112].

Acute promyelocytic leukemia cells (HL-60) [113,114] and doxo-resistant HL-60 cells [114] capitulated to juglone’s activity. On HL-60, juglone triggered ROS-mediated apoptosis. Accordingly, the antioxidant *N*-acetylcysteine (NAC) inhibited the juglone-induced programmed cell death and the modulation of crucial proteins involved in that pathway [caspases 3 and 9, poly-(ADP-ribose) polymerase (PARP), Cyt-c, diablo IAP-binding mitochondrial protein (Smac), Akt/mammalian target of rapamycin (mTor) signaling] [113]. The same mechanism of action has been highlighted for juglone in SGC-7901 human gastric cancer cells [98] and T24 bladder cancer cells [108]. In addition, on T24 cells juglone promoted endoplasmic reticulum stress through overexpression of the PKR-like ER kinase (PERK) effector eukaryotic initiation factor 2 alpha (Eif2-α), DNA damage, and lessened colony formation. As in other cell lines, juglone was tested in combination with ascorbate, resulting in a significant improvement in its antitumour activity [108].

Beside a direct eradication of cancer cells, a matching interesting therapeutic intervention could be represented by targeting fibroblasts. Fibroblasts are crucial cellular elements in tumours. They can deliver oncogenic signals, promote angiogenesis and cancer progression, and have a role in metastasis [157]. Juglone induced early DNA single-strand damage on human fibroblasts that translated in apoptosis and necrosis [158].

Numerous studies have demonstrated the ability of juglone to inhibit the PIK3/Akt cascade, but Chae et al. [101] linked the inhibition of this pathway with the ability of juglone to avoid the differentiation of normal JB6 Cl 41 skin cells under the effect of the cancer promoters 12-*O*-tetradecanoylphorbol-13-acetate (TPA) and endothelial growth factors. This suggests that juglone blocks several of the molecular pathways that are involved in cancer development.

#### 3.1.4. Peptides

Due to their fat, protein, vitamin, and mineral content, walnut kernels are a very healthy source of nutrients. For many years, they have been employed for their perfect balance of ω-3 and ω-6 PUFAs [23]. Oils are extracted by discarding the derived residue. In part to avoid waste, but also due to the new knowledge about the anticancer potential of food-derived peptides, the residues have, in recent years, started to be used for protein extraction [116]. A large amount of evidence revealed that peptides might play a role as therapeutic agents. Indeed, some peptide sequences showed interesting antitumour potential together with minimal adverse immunogenicity and exceptional tissue permeability. Furthermore, they usually require only low-cost manufacturing procedures, and are very easy to process and modify in order to ameliorate both stability and biological activity [159]. After extraction, walnut-derived proteins have to be physiologically or artificially digested, generating the bio-functional peptides. Bioactive peptides need to be released after enzymatic hydrolysis [160,161]. Clearly, from the same protein source, different enzymes or different hydrolytic conditions, such as temperature, the enzyme-substrate ratio, or length of procedure, produce peptides of different quality and displaying different bioactivities [116]. For the first time, in 2015, a bio-peptide from walnut residual proteins showed marked anticancer potential. Since then, three promising peptide sequences have been identified. A peptide fraction obtained from chymotrypsin hydrolysis of the protein residue of walnut inhibited the survival of breast and colon cancer cells (MDA-MB231 and HT-29), an effect associated with its antioxidant activity [118]. At the same time, no toxicity was registered on the non-transformed HUVEC cells treated with the same fraction [118]. A so-called pepsin-colorase pp and a pepsin-neutrase hydrolysate showed cytotoxic activity on breast cancer cells (UACC-62), while not exerting any biological activity on a panel of cells including MCF-7, HT-29, and U251 [117]. As expected, the starting non-hydrolyzed proteins did not exert any antitumour effect [117]. So far, the most characterised peptide is the sequence CTLEW (Cys-Thr-Leu-Glu-Trp) (Figure 1), obtained through a papain enzyme reaction of walnut protein residue. Its amphiphilic structure, and the resulting stabilisation achieved thanks to the di-sulfur bonds between the side C and W, make it very suitable for crossing cell membranes. CTLEW promoted apoptosis and autophagy on MCF-7 and cell-cycle arrest on Caco-2 and HeLa cells, but did not exert any toxic effect on the non-transformed IEC-6 cells, nor on spleen lymphocytes [116]. No less important, Ma et al. demonstrated that CTLEW possesses immunomodulatory potential. It enhanced the proliferation of spleen lymphocytes and interleukin-2 (IL-2) secretion and promoted phagocytosis and nitric oxide production in macrophages [116]. In a historical moment where anticancer research is headed towards immunotherapy, this finding is of great interest.

#### 3.1.5. Extracts

The anticancer potential of various extracts originating from walnut seeds, bark, root bark, and leaves has been explored. Each mixture was prepared following different procedures; thus, even extracts obtained with the same solvents of the same part of the plant might encounter different outcomes. To make comparisons even harder, differences among the same extracts can occur according to walnut genotype [162] or to the period of drug harvesting and ripeness status. For instance, young walnut leaves store significantly higher quantities of phenolic compounds than mature leaves [122,163]. In an attempt to simplify this, all the extracts fall into two broad categories: protic or aprotic. This division clearly mimics the solvents used for the extractions themselves: methanolic mixtures will be enriched in polyphenols, whereas ether petroleum or chloroform ones will retain substances such as fatty acids [164]. In the above sections, we looked through polyphenols and their activities, while no mention of fatty acids was made. As already mentioned, walnuts contain a perfect proportion of ω-3 and ω-6 PUFAs [165]. Both classes are needed for physiologic cell growth and repair and to build other fatty acids (e.g., arachidonic acid). Experimental and epidemiologic studies demonstrate that a correct ratio of these two categories is protective for some types of cancer [165,166,167]. Some ω-3 and ω-6 PUFAs cannot be synthesised de novo, and have to be introduced through diet [168]. The most studied essential fatty acids are the ω-6 LNA and the alpha-LNAs and the ω-3 alpha LA (ALA). Dark leafy vegetables, plant oils, seeds and, remarkably, walnuts are the major dietary source of essential PUFAs [165].

Among *Juglans regia* seeds, green husk, and leaves, the latter seem to carry the highest antitumour potential [120]. Methanol extracts of these parts obtained with the same procedures were tested on human renal epithelial cells (A498 and 769-P) and colorectal cells (Caco-2). Seed extracts did not exert any effect on 769-P nor Caco-2 cells, and green husk failed only on Caco-2 cells, while leaf extract promoted cancer cell death on all tested cell lines and with the lowest IC_50_ (Table 1). The anticancer effect was not correlated with the total phenolic content in the extracts, since seeds were shown to be the most enriched in those antioxidants [120]. Other different extracts of walnut leaves have been examined for their anticancer potential on oral squamous (BHY), breast (MCF-7), and colorectal (HT-29) carcinomas. Protic and aprotic solvents with different polarities were used to prepare the mixtures (methanol, ethyl acetate, chloroform, and hexane). The most active turned out to be the chloroform one [122]. Cell-cycle arrest in the G0/G1 phase and apoptosis were responsible for the observed proliferation inhibition [122]. Furthermore, the same extract was characterised, and 5-hydroxy-3,7,4′-trimethoxyflavone, lupeol, daucosterol, 4-hydroxy-a-tetralone, *β*-sitosterol, 5,7-dihydroxy-3,4′-dimethoxyflavone, and regiolone were identified but not quantified. They were tested singularly on MCF-7 and BHY cancer cell lines and on normal mouse embryonic fibroblasts (NIH-3T3). 5,7-dihydroxy-3,4′-dimethoxyflavone and regiolone showed the best pharmaco-toxicological profile, since they promoted cell death in the two cancer cell lines (IC_50_ ranging between 21.30 to 50.98 μM) (Table 1), but were almost inactive on the normal cells treated with IC_50_ equivalent concentrations of each compound [169].

The chloroform and ethyl acetate fractions of a methanol extract and an aqueous-methanol extract of walnut showed interesting antiproliferative activity on different cell lines (WRL, HEP-G2, KB and Caco-2), with IC_50_ ranging from 9 μg/mL to 70 μg/mL (Table 1), while no effects where registered on MCF-7 cells [125]. Among these three mixtures, the most promising were the two fractions, and between them, the chloroform one showed the highest cytotoxicity. Taking a closer look, the antiproliferative activity was again inverse proportional to the total phenolic content and the antioxidant capability [125].

A different methanol extract produced by Le et al. showed slight different behavior. It induced cell death in MDA-MB231, MCF-7, and HeLa cells. Furthermore, on MDA-MB231 it led to apoptosis thanks to its ability to disrupt mitochondrial functions and promote cell-cycle arrest in S and G2/M phases [35]. The authors ascribed the cytotoxicity to two ETs, tellimagrandin I and II, since in their experiments, they induced cell death at the same extent to that triggered by the extract, and at a higher extent than all the other compounds identified in the mixture [35]. However, it’s important to highlight the fact that the single compounds were tested at higher concentrations than those found in the mixture, thereby instilling curiosity about their effect at lower concentrations, and hinting at a possible additive effect of multiple compounds in the extract.

A third methanol extract showed interesting anticancer potential on colorectal CSCs (CCSCs), isolated from HCT116. Notch-activated genes and the Wnt/β-catenin cascade drive tumour growth through CCSCs expansion [170,171]. For its part, glycogen synthase kinase 3β (GSK3β) modulates intracellular levels of β-catenin through proteasomal degradation and ubiquitination [172]. The walnut (shelled kernels) methanol extract reduced cell viability, promoted cell differentiation, and suppressed the self-renewal capacity of CCSCs, through the downregulation of delta like non-canonical Notch ligand 1, Notch1 and the modulation of the β-catenin pathway [112]. The promotion of CSC differentiation is an interesting antitumour strategy, since it leads to the suppression of the stemness status that, in turn, translates into replication arrest and generation of specialised cells [156]. (+)-Cathechin, chlorogenic acid and EA were identified as the main components of the extract, but their individual activity was not as effective as the entire mixture [123]. A lipid extract obtained from the fruit exhibited exactly the same activity on CCSCs: inhibition of CCSCs cloning and down-regulation of Notch1 and β-catenin expression and GSK3β phosphorylation [124]. ALA, LNA, a mix of PUFAs, and *γ*-tocopherol were the major components of this extract [124]. It would be interesting to compare the composition of the mixtures in order to target the common compounds that promoted the biological effect, or alternatively, to investigate their additive or synergistic effect.

An oily extract, enriched in ALA and *β*-sitosterol, decreased MCF-7 cell proliferation. Even the two single fatty acids alone affected cell viability, but through a different pattern. The entire oil triggered especially the farnesoid X receptor (FXR) [168], a nuclear receptor that promotes apoptosis and decreases aromatase activity when triggered [173,174]. Not surprisingly, its expression is correlated with that of ERs [175]. FXR was activated from ALA too, but with the addition of an intense activation of the peroxisome proliferator-activated receptor (PPAR)/retinoid X receptor (RXR). When both were activated, FXR and PPAR/RXR form a heterodimer that, in turn, plays a role in cancer growth inhibition [176].

Chloroform, methanol, and n-hexane extracts of root bark of *Juglans regia* promoted apoptosis, triggering both the intrinsic and the extrinsic pathways. They all increased the Bax/Bcl2 ratio, p53, caspase 3 and 8, and tumour necrosis factor alpha (TNF-α) expression, and downregulated the apoptotic upstream regulator mouse double minute 2 homolog (mdm-2) [121]. The chloroform extract was the most potent among the three [121], suggesting once again that its antitumour activity does not rely on polyphenols.

### 3.2. Animal Studies

In vitro studies are routinely used as preliminary models for evaluating the efficacy and safety of compounds with therapeutic potential. However, the information gathered from cell cultures usually gives a reductionist overview, caused by the inability to gain the contribution of in vivo microenvironment or knowledge about drug bioavailability [177]. Thus, the initial screenings are followed by preclinical animal studies before, in turn, advancing to human clinical trials [177]. We herein report animal studies dealing with the anticancer potential of the same molecules we presented above.

#### 3.2.1. Uro-A

In vivo studies confirmed the marked antineoplastic potential of Uro-A (Table 2), promoting its therapeutic use, especially against prostate cancer. Oral administration of 50 mg/kg Uro-A to Balb/c athymic mice inhibited cell growth of xenograft tumours derived from PC-3 (AR−) and C4-2B (AR+) cells. Likewise in vitro, Uro-A had a higher effect on the AR+ cells. Indeed, only 2 weeks were necessary to block C4-2B cell proliferation versus 7 weeks for PC-3. Furthermore, on the latter cell line, Uro-A downregulated only the Ki67 proliferation marker, while, in addition, on C4-2B cells, it significantly undermined Akt activity, showing a better pharmacological profile [83]. Another relevant characteristic of Uro-A is its toxicological profile. It is an exceptionally safe compound which is characterised by a No Observable Adverse Effect Level (NOAEL) higher than 3400 mg/kg/day in Wistar rats, that corresponds to more than 550 mg/kg/day human intake [178].

#### 3.2.2. Juglanin

Animal experiments confirmed the antitumour profile of juglanin emerging from in vitro studies. In male Balb/c mice bearing human breast cancer derived from injection of MCF-7 cells, 7 days of juglanin administration translated into decreased tumour volume, explained by apoptosis (activation of caspases 3 and 9) and autophagy (modulation of LC3) induction (Table 2). Consistently with previous studies on the same tumour type, juglanin promoted the phosphorylation of JNK, while general low toxicity was recorded [89]. Indeed, juglanin exhibited an exceptionally safe activity on athymic nude mice implanted with human A549 lung cancer cells. After 4 weeks of administration, juglanin reduced tumour volume and weight in a dose-dependent fashion, showing no liver or kidney toxicity, even at the highest tested dose (30 mg/kg/day). This interesting effect is the result of triggering both apoptosis and proliferation inhibition through the activation of the same pathways observed in the in vitro studies (PIK3/Akt, p38/MAPK, p53) [90].

The effect of juglanin on hairless mice subjected to UVB radiation was assessed. Juglanin suppressed the epidermal hyperplasia observed in the control group and the related inflammation. It modulated p38/JNK and PI3K/Akt-associated signaling pathways towards apoptosis induction. At the same time, cell-cycle arrest was probably involved in juglanin’s mechanism of action, since a decrease of Ki67 was recorded, together with an increase in cyclin D, cyclin-dependent kinase 1, and proliferating cell nuclear antigen expression. P53, p21, and p27 levels were increased; thus, they may represent the link between cell-cycle arrest and apoptosis. Once again, no toxicity on mice occurred, confirming the good pharmaco-toxicological profile of juglanin [91].

#### 3.2.3. Juglone

Juglone demonstrated antitumour efficacy in in vivo models of prostate, intestinal, and Ehrlich ascites carcinoma [106,115,179,180,181]. Juglone 200 ppm was fed to weanling F344 male rats concurrently with the induced initiation phase of carcinogenesis. It significantly reduced the incidence and multiplicity of intestinal tumours compared to animals treated with only the carcinogen [179]. A study carried out in 1967 already showed juglone’s ability to produce mitotic abnormalities on proliferative tumour cells, and to decrease the amount of ascitic fluid in Swiss/HaICR mice bearing Ehrlich ascites tumours [180]. Later, juglone was found to inhibit tumour growth and increase survival in Balb/c mice bearing Erlich carcinoma [115]. It promoted apoptosis and cell-cycle arrest, as well as oxidative stress and DNA damage. All these effects were highly potentiated by the co-administration with ascorbate. Together, they promoted lipid peroxidation, protein carboxylation, SOD increased activity, and GSH consumption, all markers of oxidative stress. At the same time, juglone intercalated DNA, but only in association with ascorbate was it able to damage nucleic acids and promote the phosphorylation of γ-H2AX. Ascorbate potentiated juglone-mediated cell-cycle arrest and apoptosis, increasing the number of cells in G1 and the expression of cell-cycle inhibitors like p53 and p16. Furthermore, only in the presence of ascorbate, juglone was able to reduce the expression of cyclin A. Apoptosis-wise, the presence of ascorbate made possible to detect cleaved PARP, and increased the Bax/Bcl2 ratio [181].

Hypoxia-inducible factor (HIF-1α) is an oxygen-dependent factor whose activation promotes the gene expression of critical factors involved in chemoresistance, such as angiogenetic factors or glycolytic proteins like glucose transporters (GLUTs) [182]. GLUT1, in turn, is involved in glycolytic metabolism by reducing glucose cell uptake. The combination of juglone and ascorbate decreased HIF-1α and GLUT1 levels, and inhibited glucose uptake [181]. Bearing in mind that tumour cells exhibit high levels of glycolysis despite the presence of oxygen [183], glucose uptake represents an important target for antitumour agents.

Taken together, these results indicate a very intriguing anticancer potential of juglone.

#### 3.2.4. Phenolic Extract

Chronic intestinal inflammations and colitis may result in colon cancer. An enriched polyphenolic extract significantly reduced tumour development and tumour size by attenuating the inflammation linked with colitis in a murine model. Tumourigenesis prevention was ascribed to the inhibition of TNF-α-induced NF-κB signaling that, in turn, attenuated acute and chronic colitis [184].

#### 3.2.5. Walnut Intake in Animal Studies

Although walnuts have a high fat content, those fats are mainly polyunsaturated, and the effects of whole walnuts are beneficial. Consumption of long chain ω-3 fatty acids, such as ALA, can reduce tumour growth or cell proliferation. Incorporation of eicosapentaenoic acid and docosahexaenoic acid (DHA) reduced inflammation or inflammatory cytokine expression that in turn provoked the inhibition of cancer cell proliferation, and the increase in cell death. Together, these events are considered at the basis of the mechanisms of action of ω-3 PUFAs [185].

A walnut-enriched diet has been tested on many mouse cancer models, and the results of all studies are almost consistent. Prostate and mammary carcinomas are the two tumours on which the antitumour activity of walnut fats was demonstrated (Table 2). In general, what all these studies proved is that walnut-fed mice are characterised by the inhibition of tumour initiation and slower tumour growth rate and size [186,187,188,189,190]. These effects even passed down from mother to pups. If walnuts were administered after weaning, they decreased breast tumour incidence on both mother and offspring in SV129 mice compared to control groups [191]. A common mechanism of action has not been discovered yet. In TRAMP mice, walnut-rich diets promoted the downregulation of the insulin-like growth factor (IGF-1) [185,188], a protein directly linked to the risk of prostate and breast cancer development [192]. In contrast, no IGF-1 modulation was recorded in HT-29 xenografted mice [189]. In this model, walnuts provoked a decrease in VEGF and angiogenesis. Central areas of necrosis were found in the tumours of sacrificed mice. This information confirmed that the impaired ability to maintain sufficient oxygen and nutrient supply via bloodstream caused a reduction in tumour growth [189]. Of note, in mammary carcinoma models, walnut consumption provoked a rearrangement in the fat composition of tumour cell membranes, enrichment in ω-3 fatty acids and in ALA, DHA, and circulating high-density lipoprotein content [186]. The direct hypothesised consequence was that in this way, walnuts lessened inflammation and/or decreased the expression of inflammatory cytokines, resulting in slower tumour proliferation [186]. All the mentioned studies tried to understand which compounds within walnuts were able to promote their antitumour activity; many of them demonstrated that the observed cancer risk reduction cannot be explained exclusively by the ω-3 content of the diet [185,188], but by a synergistic activity of many compounds. However, in mice bearing HT-29 tumours, the activity of walnut overlapped with that of flaxseed [189], suggesting the need of further studies to assess the basis of the antitumour potential of walnut intake.

## 4. Human Studies

Only a few studies have explored the anticancer effects of *Juglans regia* in humans, and those covered only the relationship between chronic walnut consumption and the expression of specific cancer biomarkers. PSA, as previously mentioned, is a very common biomarker for prostate cancer. In particular, the ratio between PSA free form and its total content (the percentage of free PSA: PFP) is used to specifically diagnose prostate cancer by avoiding false positives [195,196]. Spaccarotella et al. [196] discovered that consuming 75 g/day of walnut for 8 weeks did not reduce PSA expression, even if a non-significant increase in PFP was recorded. Furthermore, they demonstrated that daily walnut intake increased serum *γ*-tocopherol levels, demonstrating that it was not enough to trigger a chemopreventive effect [196]. Simon et al. [197] prolonged the administration of walnuts to participants for a total of 6 months and focused their attention on ALA intake. Thanks to a walnut-rich diet, ALA intakes increased by more than three times compared to the control group. However, no effect was detected on PSA serum levels [197].

It’s noteworthy, however, that both studies pointed their attention on a very specific target. Since the data presented in this review show that walnut’s mechanism of action is tumour-specific, further study will be necessary to assess its global antitumour potential. At the same time, in vitro and animal studies should be taken into consideration in order to build on the most promising compounds and extracts.

## 5. Conclusions

Nowadays, natural compounds play a crucial role in anticancer therapy, either as whole or single molecule. Epidemiologic studies showed that a plant-enriched diet lowers the risk of many chronic diseases, including cancer [198]. For its part, *Juglans regia*, better known as common walnut, demonstrated interesting antitumour potential on different cell lines and animal models. Both single molecules stored in different parts of the plant and fully-fledged extracts exhibited interesting anticancer potential. The present review outlined the phytochemical composition of walnut and its resulting antitumour potential. Walnut is a container of very active molecules, such as EAs, quinones, and fatty acids. The compounds discussed in this review owe their potential antitumour activity to different mechanisms, which can be summarised as cell-cycle arrest, apoptosis induction, and metastatisation inhibition. Many molecular mechanisms are involved in this phenomenon. Uro-A, juglone, juglanin, and walnut-extracted peptides exhibit unquestionable antitumour activity on different in vitro tumour types confirmed by in vivo studies. Of note, looking at a possible clinical translation, juglone showed even better cytotoxic activity than tamoxifen, the gold standard drug for ER+ breast cancer therapy, on MCF-7 cells [93]. However, a crucial issue that impedes the elaboration of an exhaustive picture of the antitumour potential of *Juglans regia* is the scarcity of clinical studies. To our knowledge, the only works that linked cancer therapy and walnut intake are summarised here, and both suggest that 6 months of chronic intake is unable to reduce the expression of cancer biomarkers. Certainly, further studies are needed to draw convincing conclusions about walnut activity on humans.

As mentioned, natural products are often proposed in the oncological field to potentiate the cytotoxic activity of traditional anticancer agents and reduce their toxicity. Uro-A sensitised colon cancer cells to the gold-standard drug 5FU and its metabolite [66], while juglanin synergised the effect of doxo on lung cancer cells and doxo-resistant cancer cells [133]. Furthermore, the safety profile of Uro-A and juglanin has been shown, thanks to both in vitro and in vivo experiments. The major safety concern is represented by the mutagenic activity of the allelopathic juglone, that already scared our ancestors. Juglone has been shown to promote DNA damage, emerging as a mutagenic substance. However, in itself, this fact is not alarming. Many traditional anticancer drugs efficiently target DNA to kill tumour cells. Actually, as with all therapeutic agents, ensuring an appropriate risk-benefit assessment will be necessary. Alongside, juglone mutagenic activity does not necessarily translate into walnut mutagenic activity. To evaluate the potential risk for human health deriving from walnut dietary intake, the so-called matrix effect has to be taken into consideration. Plants are complex structures that store many compounds that generate a certain effect, due to synergistic activities. This means that the final effect of the mixture will not exactly mirror the sum of the effects of single compounds. For example, antioxidants and other chemopreventive substances can quench the harmful effect of others molecules [199]. As the phytochemical composition has shown, this is certainly the case with walnuts. Indeed, all the studies involving walnut extracts or walnut-enriched diets disclosed a negligible toxicity together with antimutagenic activity and selective effect towards tumour cells. In contrast, different studies regarding walnut-enriched diets showed beneficial properties, such as prevention or delay of tumour initiation.

In conclusion, what is certain is that the antitumour potential of walnut finds a solid foundation in its intrinsic chemical composition, but further studies are needed to identify the best approach to exploit this potential, and to confirm this activity on humans, considering both efficacy and safety.

## Figures and Tables

**Table 1:** In vitro pharmacological activities of extracts and compounds isolated from *Juglans regia*.

Compound or Extract	Cell Line	IC_50_ or Concentration Range (μM) ^a^	Cell-cycle Inhibition Phase and Markers	Apoptosis Markers	Inhibition of Tumour Invasion and Metastasis Markers	Other Mechanisms and Markers	Reference
Juglanin	MCF-7	IC_50_ 24 h: 26.35	G2/M	⬇ Bcl2, ⬆ Bad		Autophagy: formation of autophagosome, ⬆ LC3B-II	[89]
		IC_50_ 48 h: 14.38					
	SKBR3	IC_50_ 24 h: 20.07		⬆ Bax			
		IC_50_ 48 h: 17.69		⬆ Caspase 3, 8 and 9			
				⬆ ROS			
				Chromatin condensation			
	MDA-MB231	IC_50_ 24 h: 29.13					
		IC_50_ 48 h: 23.25					
	BT474	IC_50_ 24 h: 24.17					
		IC_50_ 48 h: 19.85					
	A549	0–80		Sub-G1 cells		Autophagy: autophagic vacuoles, ⬆ LC3, ATG7 and ATG3	[90]
				Chromatin condensation and DNA fragmentation			
				⬆ PARP			
				⬆ Caspase 3, 8 and 9			
				⬇ Bcl-2 and Bcl-xl, ⬆ Bax and Bad			
				⬇ TRAIL, DR4, DR5 and FADD			
	H1975			⬆ p53			
				⬆ ROS			
				⬇ NF-kB			
				⬇ PI3K/Akt			
				⬇ MAPK and ERK1/2, ⬆ p38 and JNK			
				⬆ C-Jun			
				⬆ C-Abl			
				⬆ p73			
	HCC827						
	B16F10	0–30		⬆ PARP			[91]
				⬇ p38/JNK			
				⬇ PI3K/Akt			
				⬇ NF-kB			
				⬆ Caspase 3			
				⬆ p53, p21 and p27			
Juglone	MCF-7	0–50		⬆ Caspase 3			[92,93]
		IC_50_ 24 h: 11.99		⬇ Bcl-2, ⬆ Bax			
				⬇ ΔΨ, ⬆ [Ca^2+^], MOMP and Cyt-c			
				⬆ p53			
				⬇ p-Akt			
				⬆ ROS			
				⬇ GSH, catalase, superoxide dismutase and glutathione peroxidase			
				⬆ Lipid peroxidation			
	MCF-7 Adr	The concentrations tested were not reported.	G2/M		⬇ Migration		[94]
			⬇ Cyclin E		⬇ VEGF-A, -B and -C		
	SKBR3	0–50	G0/G1		⬇ Colony formation		[95]
	MDA-MB231	IC_50_ 24 h: 10.35	G2/M		⬇ Migration		[96]
	BxPC-3	IC_50_ 24 h: 21.05			⬇ Adhesion and cell invasion		[97]
					⬇ MMP-2 and -9		
					⬇ VEGF		
					⬇ Phactr-1		
	PANC-1	IC_50_ 24 h: 21.25			⬇ Adhesion and cell invasion		
					⬇ MMP-9		
					⬇ VEGF		
					⬇ Phactr-1		
	SGC-7901	IC_50_ 24 h: 36.52		⬆ Caspase 3			[98]
		IC_50_ 48 h: 25.38		⬇ Bcl-2, ⬆ Bax			
				⬇ ΔΨ, ⬆ Cyt-c			
				⬆ ROS			
	B16F1	IC_50_ 24 h: 7.69		⬆ Sub-G1	⬇ Colony formation	Necrosis	[99]
				Membrane blebbing		Mutagenic activity: ⬆ Micronuclei frequency	
				Chromatin condensation			
				DNA fragmentation			
				⬆ ROS			
				⬆ LDH			
	A2058	0–20		⬆ ROS			[100]
				⬆ p53			
				⬆ p38			
	MEWO	0–20		⬆ TRAIL			
				⬆ ROS			
				⬆ p53			
				⬆ p38			
	JB6 CI41	0–5		⬇ PI3K	⬇ TPA- or EGF-induced cell transformation	⬇ TPA- or EGF-induced AP-1 and COX-2	[101]
				⬇ TPA-induced activation of AKT			
				⬇ TPA-induced c-Jun and c-fos activation			
	HeLa	0–100		⬆ Caspase 3, 8 and 9			[102,103]
		IC_50_ 24 h: 33		⬆ PARP			
				⬇ Bcl-2, ⬆ Bax			
				⬆ Cyt-C			
				⬆ Fas and FasL			
				⬆ p-JNK			
	SKOV3	IC_50_ 24 h: 30.13	G0/G1	⬆ Caspase 3, ⬇ Procaspase 9	⬇ MMP-2		[104]
			⬇ Cyclin D1	⬇ Bcl-2, ⬆ Bax			
				⬆ Cyt-c			
	LNCap	IC_50_ 24 h: 13.8–32.2		Chromatin condensation, cell shrinkage and membrane blebbing	⬇ EMT	⬇ PSA	[105,106,107]
		IC_50_ 48 h: ≃15		⬆ Caspase 3 and 9	⬆ E-cadherin, ⬇ N-caderin and vimentin	⬇ AR	
				⬇ ΔΨ	⬇ Akt/GSK-3β/Snail		
	LNCap-AI	IC_50_ 24 h: 43.1					[107]
	DU145	IC50 48 h: ≃10					[106]
	T24	IC_50_ 24 h: ≃28.5		⬆ Caspase 3	⬇ Cell motility	DNA damage: ⬆ γ-H2AX	[108]
				⬆ PARP		ER stress: ⬆ PERK and Eif2-α	
	C6	0–64	G0/G1	⬆ ROS	⬇ Cell spheroid invasiveness		[109,110]
		IC_50_ 24 h: ≃10.4		⬇ MRC complex 1	⬇ Metastasis formation		
	U251	0–20		Chromatin condensation	⬇ Migration		[111]
				⬆ Caspase 3	⬇ Angiogenesis		
				⬇ TGFβ1/Smad/miR-21			
				⬇ Pin1			
	U87	0–40		⬆ Caspase 9			[112]
	SHG62			⬆ ROS			
	SHG66			⬆ p38/MAPK			
	HL-60	IC_50_ 24 h: ≃8		⬆ Caspase 3 and 8			[113]
				⬆ PARP			
				cc Cyt-c			
				⬆ ROS, ⬇ GSH			
				⬆ Smac			
				⬇ Akt/mTor			
	HL-60 doxo-resistant			⬆ Oxygen consuption			[114]
				⬆ Quinone reductase activities			
				⬆ Superoxide dismutase and glutathione S-transferase			
Juglone + Ascorbate (1 mM)	MCF-7	0–50		DNA fragmentation		DNA damage: ⬆ γ-H2AX	[93,115]
		IC_50_ 24 h: 28		⬇ Bcl-2, ⬆ Bax		Necrosis	
				⬆ ROS			
				⬇ Catalase and glutathione peroxidase			
				⬆ Foxo3a and Foxo1			
				⬇ p-Akt			
	T24	IC_50_ 24 h: 6.3		⬆ ROS, ⬇ GSH	⬇ Cell motility	DNA damage: ⬆ γ-H2AX	[108]
						ER stress: ⬆ PERK, and eif2-α	
Uro-A	MCF-7	IC_50_ 48 h: 95.56					[78]
	A549	IC_50_ 48 h: 17.81					
	HepG2	0–200	G2/M	⬆ Caspase 3			[51,78]
		IC_50_ 24 h: 137	⬇ Cyclin D1	⬆ Bax			
		IC_50_ 48 h: 40.53		⬆ p53			
				⬇ c-Myc			
				⬆ p38/MAPK			
				⬇ TCF/LEF			
				⬇ β-catenin			
				⬇ IL6 and IL1β			
				⬇ NF-kB,			
				⬇ COX-2 and iNOS			
				⬆ PUMA and NOXA			
	HepG2.2.15	IC_50_ 24 h: >120		⬆ Caspase 3			[67]
				⬇ Bcl-2, ⬆ Bax			
				⬇ Lin28a, ⬆ Let-7a			
				⬇ HMGA2 and K-Ras			
				⬇ Sp-1 and Zcchc11			
	ECC-1	0–50	G2/M				[88]
	HEC1A		⬆ Cyclin B1 and E2				
			⬆ p-cdc2 and cdc25B				
			⬆ Myt1				
	Ishikawa		⬆ p21	⬇ ER-α ans GRIP1, ⬆ ER-β, PGR, pS2 and GREB1			
	LNCap	0–40	G0/G1	⬆ Caspase 3 and 7		⬇ PSA	[36,37,82]
		IC_50_ 24 h: 13.8		⬇ Bcl-2		⬇ AR	
				⬆ CDKN1A			
				⬇ Fibronectin-1			
				⬆ p21			
	PC3	0–200		⬆ Caspase 3			[37,83]
		IC_50_ 24 h: 70		⬆ PARP			
	C4-2B	0–200		⬆ PARP		⬇ PSA	[83]
		IC_50_ 24 h: 35		⬆ Caspase 3		⬇ AR	
				⬇ p-Akt			
	DU145	IC_50_ 24 h: 33.4					[36]
	UMUC3	IC_50_ 48 h: 23.92	G2/M	⬇ PI3K/Akt			[78]
				⬇ ERK 1/2			
				⬇ SAPK/JNK			
	T24	IC_50_ 48 h: 43.90		Chromatin condensation ⬆ Caspase 3 ⬆ PPAR-γ ⬆ p38/MAPK ⬇ MEKK1 and c-Jun			[79]
	Caco-2	0–100		⬆ C-Myc			[52,55,58,66]
		IC_50_ 24 h: 87		⬆ DUSP6			
		IC_50_ 48 h: 42.80–81	S	⬆ Fos			
	SW480	0–100	G2/M	⬆ C-Myc			[52,58,66]
		IC_50_ 48 h: 59.45	⬆ Cyclin A and B1	⬆ CDKN1A			
				⬆ CTMNBI			
				⬆ EGF3			
	HT-29	0–100	S				
		IC_50_ 48 h: 46.01	G2/M				
	SW620	IC_50_ 24 h: ≥15	G2/M	⬆ Caspase 3	⬇ Cell migration	Autophagy: ⬆ LC3	[64]
					⬇ MMP-9 activity		
Uro-A + Uro-B	LNCaP	20 + 20		⬇ Bcl-2		⬇ PSA	[37]
	PC3					⬇ AR	
Uro-A + Uro-C + EA	Caco-2	85 + 10 + 5	S	⬆ p53			[52]
Iso-Uro-A + Uro-A + Uro-B +Uro-C + AE		50 + 30 + 10 + 5 + 5	G2/M	⬆ K-ras			
CTLEW	MCF-7	IC_50_ 48 h: 1.620 μg/mL		⬆ Sub-G1		Autophagy: ⬇ LC3-I and ⬆ LC3-II	[116]
				Phosphatidilserine externalisation			
	Caco-2	IC_50_ 48 h: 650 μg/mL					
	HeLa	IC_50_ 48 h: 600 μg/mL					
Peptide from walnut pepsine-colorase pp hydrolysis	UACC-62	IC_50_ 24 h: 0.25 μg/mL					[117]
	U251	IC_50_ 24 h: >250 μg/mL					
	MCF-7						
	NCI-adriamycin resistant						
	786-O						
	NCI-H460						
	PC3						
	OVCAR-03						
	HT-29						
	K562						
Peptide from walnut pepsine hydrolysis	UACC-62	IC_50_ 24 h: 710 μg/mL					
	U251	IC_50_ 24 h: > 250 μg/mL					
	MCF-7						
	NCI-adriamycin resistant						
	786-O						
	NCI-H460						
	PC3						
	OVCAR-03						
	HT-29						
	K562						
Peptide from walnut neutrase hydrolysis	UACC-62	IC_50_ 24 h: 25 μg/mL					
	U251	IC_50_ 24 h: >250 μg/mL					
	MCF-7						
	NCI-adriamycin resistant						
	786-O						
	NCI-460						
	PC3						
	OVCAR-03						
	HT-29						
	K562						
Peptide from walnut chymotrypsin hydrolysis	MDA-MB231	IC_50_ 24 h: 650 μg/mL					[118]
Chloroform green husk extract	PC3	IC_50_ 24 h: 91.14 μg/mL		⬆ Caspase 3 ⬇ Bcl-2, ⬆ Bax ⬆ p53			[119]
		
		
		
N-hexane green husk extract		IC_50_ 24 h: 27.29 μg/mL					
		
		
		
Methanol green husk extract		IC_50_ 24 h: 66.72 μg/mL					
		
		
		
	A-498	IC_50_ 24 h: 285 μg/mL					[120]
	769-P	IC_50_ 24 h: 496 μg/mL					
	Caco-2	IC_50_ 24 h: > 500 μg/mL					
Chloroform root bark extract	MDA-MB231	0–50 μg/mL		⬆ Caspase 3 and 8			[121]
				⬇ Bcl-2, ⬆ Bax			
N-hexane root bark extract				⬆ p53			
				⬆ TNF-α			
Methanol root bark extract				⬇ Mdm-2			
Methanol leaf extract	A-498	IC_50_ 24 h: 226 μg/mL					[120]
	769-P	IC_50_ 24 h: 352 μg/mL					
	Caco-2	IC_50_ 24 h: >500 μg/mL					
Chloroform fraction of aqueous-ethanol leaf extract	MCF-7	IC_50_ 24 h: 500 μg/mL	G0/G1	⬆ Sub-G1			[122]
		IC_50_ 48 h: 360 μg/mL					
	HT-29	IC_50_ 24 h: 810 μg/mL					
		IC_50_ 48 h: 530 μg/mL					
	BHY	IC50 24 h: 590 μg/mL					
		IC_50_ 48 h: 450 μg/mL					
N-hexane fraction of aqueous-ethanol leaf extract	MCF-7	IC_50_ 24 h: >1500 μg/mL					
	HT-29	IC_50_ 48 h: >1500 μg/mL					
	BHY						
Methanol fraction of aqueous-ethanol leaf extract	MCF-7						
	HT-29						
	BHY						
Ethyl acetate fraction of aqueous-ethanol leaf extract	MCF-7	IC_50_ 24 h: 1060 μg/mL					
		IC_50_ 48 h: 520 μg/mL					
	HT-29	IC_50_ 24 h: 1490 μg/mL					
		IC_50_ 48 h: 1060 μg/mL					
	BHY	IC_50_ 24 h: 1410 μg/mL					
		IC_50_ 48 h: 820 μg/mL					
Methanol fruit extract	CSCs	0–40 μg/mL			⬇ Formation of colonies and spheres	⬆ CK20	[123]
						⬇ Notch 1	
						⬇ DLK1	
						⬇ β-catenin	
						⬇ p-GSK3β	
	Primary human colorectal cancer cells					⬇ Notch 1	
						⬇ DLK1	
Chloroform-methanol fruit extract	CSCs	0–1000 μg/mL			⬇ Colony formation	⬇ β-catenin	[124]
						⬇ p-GSK3β	
						⬇ Notch 1	
Methanol fruit extract	MCF-7	IC_50_ 24 h: 348 μg/mL					[125]
	WRL-68	IC_50_ 24 h: 301 μg/mL					
	HepG2	IC_50_ 24 h: 405 μg/mL					
	Caco-2	IC_50_ 24 h: 305 μg/mL					
	KB	IC_50_ 24 h: 403 μg/mL					
Aqueous methanol fruit extract	MCF-7	IC_50_ 24 h: >500 μg/mL					
	HepG2	IC_50_ 24 h: 66 μg/mL					
	WRL-68	IC_50_ 24 h: 55 μg/mL					
	Caco-2	IC_50_ 24 h: >500 μg/mL					
	KB	IC_50_ 24 h: 251.6 μg/mL					
Chloroform fraction of aqueous-methanol fruit extract	MCF-7	IC_50_ 24 h: >500 μg/mL					
	WRL-68	IC_50_ 24 h: 60.6 μg/mL					
	HepG2	IC_50_ 24 h: 9 μg/mL					
	Caco-2	IC_50_ 24 h: 35.66 μg/mL					
	KB	IC_50_ 24 h: 40 μg/mL					
Methanol-soluble fraction of aqueous-methanol fruit extract	MCF-7	IC_50_ 24 h: 350 μg/mL					
	HepG2	IC_50_ 24 h: 351.6 μg/mL					
	WRL-68	IC_50_ 24 h: 455μg/mL					
	Caco-2	IC_50_ 24 h: 301 μg/mL					
	KB	IC_50_ 24 h: 351.6 μg/mL					
Methanol-insoluble fraction of aqueous-methanol fruit extract	MCF-7	IC_50_ 24 h: 500 μg/mL					
	HepG2	IC_50_ 24 h: 298.3 μg/mL					
	WRL-68	IC_50_ 24 h: 351 μg/mL					
	Caco-2	IC_50_ 24 h: 356.6 μg/mL					
	KB	IC_50_ 24 h: 353 μg/mL					
N-hexane fraction of aqueous-methanol fresh fruit extract	MCF-7	IC_50_ 24 h: 403 μg/mL					
	HepG2	IC_50_ 24 h: 301.6 μg/mL					
	WRL-68	IC_50_ 24 h: 255 μg/mL					
	Caco-2	IC_50_ 24 h: 301.6 μg/mL					
	KB	IC_50_ 24 h: 201.6 μg/mL					
Ethyl acetate fraction of aqueous-methanol fresh fruit extract	MCF-7	IC_50_ 24 h: 448.3 μg/mL					
	HepG2	IC_50_ 24 h: 15.3 μg/mL					
	WRL-68	IC_50_ 24 h: 70 μg/mL					
	Caco-2	IC_50_ 24 h: 200 μg/mL					
	KB	IC_50_ 24 h: 50.3 μg/mL					
Methanol seed extract	A-498	IC_50_ 24 h: 291 μg/mL					[120]
	769-P	IC_50_ 24 h: >500 μg/mL					
	Caco-2						

^a^ If the IC_50_ value was not specified. ⬆: Upregulation; ⬇: Downregulation; ΔΨ: Mitochondrial potential.

**Table 2:** In vivo pharmacological activities of extracts and compounds isolated from *Juglans regia*.

Compound/Diet	Experimental Model	Treatment Doses	Anticancer Effects	Molecular Targets	References
Juglanin	MCF-7-xenografted male BALB/c-nude mice	0–10 mg/kg/day (7 days)	⬇ Tumour growth	⬆ Caspase 3, 9	[89]
				⬆ LC3B	
				⬆ p-JNK	
	A549-xenografted athymic nude mice	0–30 mg/kg/day (28 days)	⬇ Tumour volume	⬆ Caspase 3	[90]
			⬇ Tumour weight	⬆ PARP	
				⬇ Bcl-2, Bcl-xl, ⬆ Bax, Bad	
	
				⬆ p53	
				⬆ TRAIL, DR4, DR5 and FADD	
				⬆ PI3K, Akt, and p-ERK1/2	
				⬆ p-p38	
				⬆ LC3BI/II, ATG7, Beclin1 and PIK3C3	
	Hairless mice subjected to UVB radiation	0–20 mg/kg/2 days per week (10 weeks)	Suppression of epidermal hyperplasia and inflammatory cell infiltration	⬇ Ki67	[91]
				⬇ p38/JNK	
				⬇ PI3K/AKT	
				⬇ IL-1β, TNF-α, IL-6	
				⬇ Cyclin D1, CDK1, PCNA	
				⬆ p53, p27, p21	
				⬆ PARP	
				⬆ Caspases 3 and 8	
Juglone	Female BALB/c-nu mice implanted with U87 stem-like cells	1 mg/kg/ day per 3 days (5 administrations)	⬇ Tumour growth		[112]
			⬆ Survival		
	MDA-MB231-xenografted nude mouse	10–40 mg/kg/day every 3 days (5 administrations)	⬇ Tumour growth		[96]
	Inbred C57BL/6J mice implanted with B16F1	1 mg/kg/day 1, 3 and 5 (3 administrations)	⬇ Tumour growth		[193]
			⬆ Survival		
	Weanling male F344 rats treated subcutaneously injections of azoxymethane	200 ppm/once per week (3 weeks)	⬇ Incidence and multiplicity of intestine tumours		[179]
	Ehrlich ascites tumour xenografted swiss/HaICR mice	0–2 mg (single injection)	Mitotic abnormalities		[180]
			⬇ Amount of ascitic fluid		
Juglone + Ascorbate	Ehrlich carcinoma- xenografted male BALB/c inbred mice	(1 mg/kg + 100 mg/kg)/day (9 days)	⬇ Tumour growth	⬆ G0/G1 cell-cycle arrest	[115,181]
			⬆ Survival	⬆ p53, p16	
				⬇ Cyclin A	
				⬆ PARP	
				⬆ Bax	
				⬇ Bcl-xL	
				⬇ HIF-α	
				⬇ GLUT1	
				⬇ GSH, ⬆ SOD	
				⬇ p-Akt	
				⬆ Protein carboxylation	
				⬆ MDA	
				⬆ γ-H2AX	
Uro A	C4-2B-xenografted male BALB/c athymic mice (nu/nu)	50 mg/kg/5 days per week (4–5 weeks)	⬇ Tumour growth	⬇ Ki67	[83]
				⬇ Akt	
	PC-3-xenografted male BALB/c athymic mice (nu/nu)			⬇ Ki67	
Walnut diet	TRAMP mice	100 g whole walnut/kg of diet ad libitum (18 weeks)	⬇ Tumour size	⬇ IGF-1	[188]
				⬇ High density lipoprotein, total cholesterol	
				⬆ Insulin sensitivity	
				⬇ Glucose-6-phosphate	
				⬇ Succinylcarnitine	
				⬇ 4-hydroxybutyrate	
				⬆ PCK1 and CIDEC	
		155 g of whole walnut/kg of diet ad libitum (9, 18, 24 weeks)	⬇ Tumour growth and size.	⬇ Plasma IGF-1	[185]
				⬇ Resistin	
				⬇ Low density lipoprotein	
	LNCaP xenografted nude mice	113 g of whole walnut/kg of diet ad libitum (126 days)	⬇ Number of tumours		[190]
			⬇ Xenografts growth		
	HT-29 xenografted female nude (nu/nu) mice	110 g of whole walnut/kg of diet (25 days)	⬇ Tumour weight	⬇ VEGF	[189]
	Pups born after female SV129 mice bred with transgenic homozygous C(3)1/SV40 T antigen SV129 male mice Female SV129	111 g of walnut/kg of diet ad libitum (optional 2 weeks before breeding + 21 days of weaning + 110, 130 or 145 days)	⬇ Tumour incidence	Altered expression of 84 genes associated with proliferation and differentiation	[191]
			⬇ Tumour Multiplicity		
			⬇ Tumour size		
	MDA-MB231-xenografted nude mice	113 g of whole walnut/kg of diet (35 days)	⬇ Tumour growth		[194]

	HT-29-xenografted athymic nude (nu/nu) mice	111 g of whole walnut/kg of diet ad libitum (25 days)	⬇ Tumour growth	⬆ ALN, eicosapentaenoic, DHA and total ω-3 fatty acids	[186]
				⬇ Arachidonic acid	
				⬇ miRNAs 1903, 467c and 3068, ⬆ miRNA 297a	

⬆: Upregulation; ⬇: Downregulation.
